# The tablet computer’s impact on learning and National Dental Examination scores in orthodontics - a mixed-method research

**DOI:** 10.1186/s13005-019-0195-7

**Published:** 2019-05-03

**Authors:** Thomas Stamm, Irina Triller, Ariane Hohoff, Moritz Blanck-Lubarsch

**Affiliations:** Department of Orthodontics, University of Münster, Albert-Schweitzer-Campus 1, 48149 Münster, Germany

**Keywords:** E-learning, Medical education, Tablet PC, Orthodontics, Dental examination score, Mobile learning

## Abstract

**Background:**

To assess the educational impact of a one-to-one tablet PC (TPC) program by analysing university students’ learning skills and related scores of the National Dental Examination (NDE) in Germany.

**Methods:**

The study design was a mixed-method approach consisting of a survey and a comparison of NDE scores. Students received a loaned non-preloaded and non-managed TPC during three consecutive orthodontic semesters. Usability and learning benefits in clinical and nonclinical settings were assessed by a survey. After the participating students had passed the NDE in a standard period of study, their grades were compared with those of students from the semester prior to TPC introduction.

**Results:**

One hundred and eight students (36 females and 72 males) received an TPC and participated in the survey. Of these, 53 passed the NDE in a standard period of study. 64 students from the semester before TPC introduction, who passed in the regular period of study, were chosen as non-TPC control group.

Survey: Students’ expectations concerning TPC benefits increased significantly after TPC usage (*P* = 0.000). TPCs were rated more useful for learning at places outside the clinic setting than for inside (*P* = 0.000). PDFs and communication applications were used more in nonclinical settings (*P* = 0.008 and 0.000, respectively). NDE scores: Concerning the examination parts relating to theoretical knowledge and clinical knowledge, students with full TPC use achieved higher scores than did those without TPC use (*P* = 0.006 and 0.002, respectively). Scores for manual skills showed no differences, neither for students with and without TPC, nor within the semester after TPC introduction (*P* = 1.000).

**Conclusions:**

This is the first study to analyse a one-to-one TPC program in the orthodontic curriculum and measure the effect of TPC usage on NDE scores. Students’ expectations concerning the TPC benefit in the orthodontic curriculum improved significantly after using the devices. We have shown that NDE scores in theoretical knowledge increased significantly after TPC deployment whereas scores in motor skills remained unchanged. The results suggest that the TPC has a positive learning effect on theoretical knowledge in orthodontics.

**Trial registration:**

Permission to conduct this study was given by the Ethics Committee of the Department of Medicine of the University of Münster, Germany (2012-12-13).

## Background

As ownership of tablet-PCs (TPCs) grows, there is increasing interest in medicine as to how they might be used for teaching and patient care. Because of their portability, connectedness, and responsiveness, TPCs like the iPad (Apple Inc., Cupertino, CA, USA) are seen to reach the goal of ubiquitous access to information at any time in the clinical environment. The ways in which TPCs are used is also expanding [1–4].

The School of Medicine at the University of California, Irvine, the Yale School of Medicine, and the Stanford University School of Medicine were the first universities to introduce TPCs for their medical curriculum [5–7]. The early goals were to save paper and to make course materials more accessible by distributing lectures and textbooks digitally. George et al. were the first authors who examined the effects of TPC use in undergraduate medical education [8]. They found that this device has value in preclinical education but does not fully replace printed hand-outs [8]. The only available survey of dental students revealed that 89% of the respondents employed laptops for digital learning and only 16% additionally use TPCs [9].

A further intention of TPC deployment is seen in the increase of physicians’ workflow efficiency [10]. It can be shown that the TPC saves time in indirect patient care, such as updating medical charts, documentation, ordering tests, and other administrative tasks. Almost 90% of surveyed residents used their TPCs for clinical responsibilities at work [10]. Lobo et al. found similar results concerning resident efficacy but examined only 12 participants [11]. A critical examination of TPC use among 115 internal medicine residents revealed that expectations decrease significantly after TPC deployment [12]. A further investigation of a small number of participants (*n* = 25) highlighted the importance of computer proficiency across users, which is a main factor in heterogeneous TPC use [13]. Lehnbom et al. measured interactions between data entry devices and the electronic health record (EHR) system [14]. They found that most clinical information was accessed from TPCs (56.2%), followed by computers-on-wheels (35.8%), and PCs (7.9%) [14].

The positive results of TPC deployment in medical education are not transferable to dental education because of the different curricula. The dental curriculum at the University of Münster, Germany, involves apart from medical and dental lectures essential laboratory and clinic time in which students practice their manual skills. By the beginning of the seventh semester, all students have passed the preclinical courses and are now fully engaged in patient care. Dental students expect to work in varied contexts. They treat actual patients in the various departments and fabricate prostheses and orthodontic appliances in the laboratory. In between patient treatment and laboratory work, students have traditional face-to-face lectures, seminars, and live demonstrations where they learn step-by-step procedures. In this mixed setting, introducing mobile learning is helpful because within each of the disciplines, students should have access to all the resources they need.

**Fig 1:**
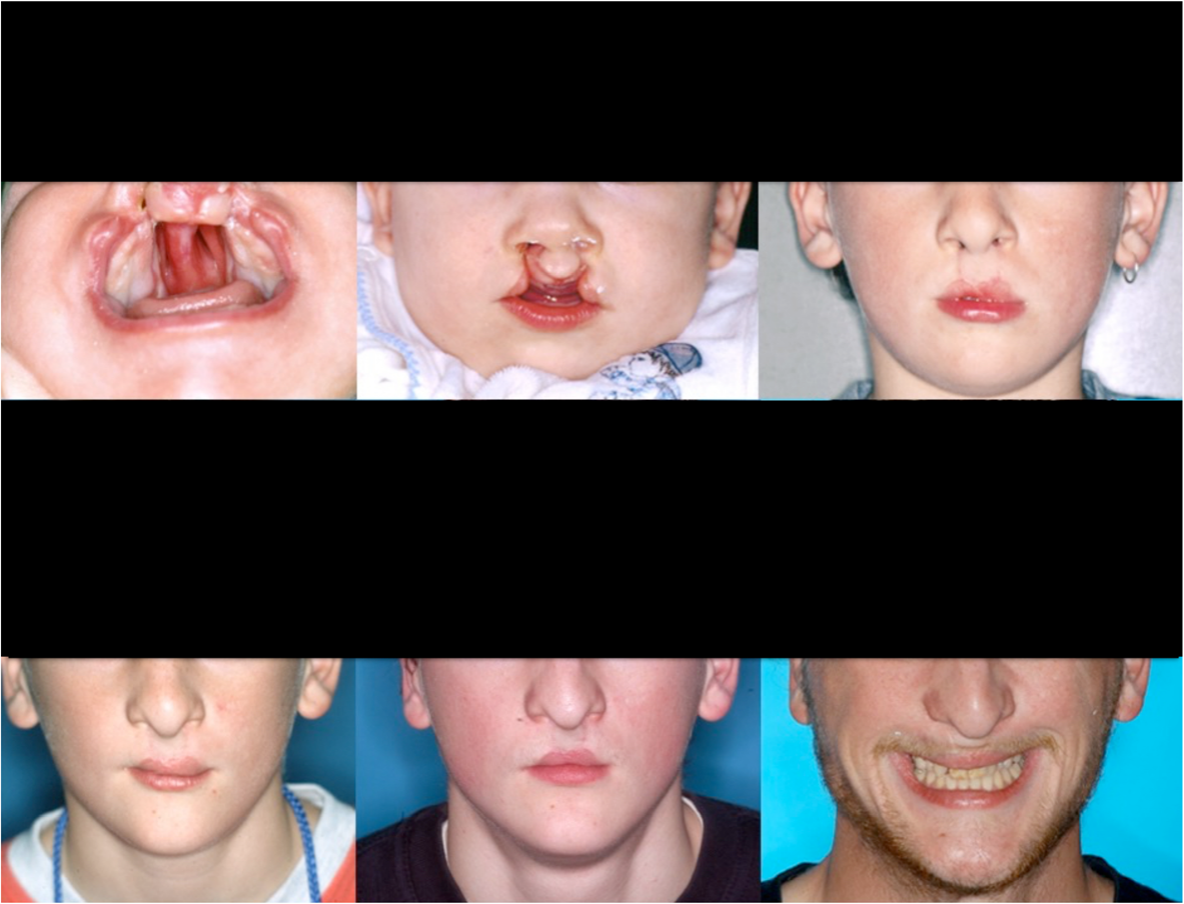
Long treatment time, e.g. of a cleft lip and palate patient, is challenging for orthodontic students. To understand treatment effects and overlaying growth and development, years of medical history from birth (top row, left) to adulthood (bottom row, right) must be reviewed

The orthodontic curriculum comprises theoretical and practical teaching modules. A main disadvantage for learners is the long treatment period, which could last for years, depending on the patient’s individual needs. Therefore, course participants see only snapshots of the whole treatment process. For example, a cleft lip and palate patient needs treatment from birth to adulthood (Fig. 1). The medical data of these patients are very extensive and are located in various databases (medical EHR, dental EHR, picture archiving and communication system, orthodontic information system).

It is difficult for a student to review all the EHR data on one patient in the treatment room in addition to the clinician’s documentation tasks. As a result, course participants are often discouraged because of the restricted access, inherent complexity of procedures, and number of details that must be learned in a limited time.

To improve the learning environment, the Department of Orthodontics at the University of Münster introduced TPCs for mobile access to case-based digital teaching information. Each orthodontic student (seventh to eighth semester) was provided with a loaned TPC, and the department was equipped with wireless local area network access points. From the variety of TPCs the iPad was chosen because of hygienic considerations in the clinic: The absence of a fan allows covering the iPad with cling film, the touch screen works faultlessly when using both a protection cling foil and medical gloves.

The intent of the one-to-one TPC program was to allow students to access all relevant (longitudinal) data of patients treated during the clinical courses. The students were free to select the sources and amounts of information they needed for their individual orthodontic learning curve. We believe that this kind of independence has a positive effect on students’ motivation and overall education quality.

Therefore, the aim of this study was twofold: first, to assess the educational benefits of TPC deployment by evaluating students’ self-reported learning skills and second, to assess a possible related effect on the National Dental Examination (NDE) scores.

## Methods

Permission to conduct this study was given by the Ethics Committee of the Department of Medicine of the University of Münster, Germany (2012-12-13). Students attending a clinical course of orthodontics at the Dental School of the University of Münster, Germany, were invited to complete a survey after deployment of a one-to-one TPC program. Each participating student of three consecutive orthodontic semester groups (S1 = semester group of TPC introduction; S2 = first and S3 = second semester group after TPC introduction), received an TPC for two semesters of their orthodontic education including semester breaks (seventh and eighth semester). The TPCs were not preloaded with content nor managed by a mobile device manager. Each student administrated his or her loaned TPC him−/herself and was free to decide how to use the device for learning and private activities. Students returned the TPC at the end of their eighth semester and completed the survey.

The grades of those students who participated in the program and finally passed the NDE in a standard period of study were assessed. All students, failing a semester at any point of their dental education were excluded from this study. Scores of the orthodontic exams from students before and after TPC deployment were compared. Students from the semester before TPC introduction (S0 = before TPC introduction) served as non-TPC control group. Teaching methods and materials were not modified during time period S0-S3 except for PDF documents being available on the TPC (S1-S3).

### Survey design

A review of the literature was conducted to identify usability topics for inclusion in the survey tool. To identify relevant articles a Pubmed search was performed with the following search strategy: “(“tablet pc”[Title/Abstract] OR ipad[Title/Abstract] OR “mobile device”[Title/Abstract] OR portable[Title/Abstract] OR smartphone[Title/Abstract]) AND (assessment[Title/Abstract] OR survey[Title/Abstract] OR questionnaire[Title/Abstract]) AND clinic[Title/Abstract]”. The search was limited to English language. The time period covered research published from January 1980 to December 2013.

The primary search resulted in 93 articles. A restriction to “dent*” instead of “clinic” revealed 48 articles. For both search results titles and abstracts were screened and 57 articles were identified for fulltext reading. Articles with a different focus than the usability of a device like psychosocial assessments, self monitoring, or questionnaires used for data recordings were excluded. Seven articles remained from which further four studies were excluded for the following reasons: a) assessment of visual and audio quality, b) assessment of different devices and operating systems concerning specific e-learning offers, c) survey items do not assess usability, and d) question items were tailored to specific non dental learning goals. Three articles [15–17] were finally selected whereas two of them used the System Usability Scale [18] which is implemented in SoSci Survey [19] as a standard questionnaire template.

We adapted the questionnaire from Reynolds et al., who compared the educational benefit and usability of mobile devices in the dental clinic and at home [16]. We customized the items under “How easy was it to use the PDA at. ..? ” concerning the requirements and resources of our department. Later, items concerned with the educational benefits for other dental courses were added.

The survey was constructed using the online tool SoSci Survey in accordance with published guidelines for the creation of online surveys and pilot tested on a small group of students two semesters before TPC deployment [19]. During this process, we modified the wording for maximum clarity and length and to ensure that the questions did not include stereotypes. The final survey was used for assessment of the two semesters of their orthodontic education.

### National Dental Examination scores

During the NDE in orthodontics, the students were examined on their theoretical knowledge, chairside clinical skills, and manual skills (appliance). Theoretical knowledge was assessed by an oral exam on general principles in orthodontics and diagnosing an orthodontic case. During the assessment of chairside clinical skills, students diagnosed an orthodontic patient, developed a treatment strategy (including the choice of appliance), and inserted a removable appliance. Manual skills were determined by assessing an orthodontic appliance fabricated by the student during the examination phase (5 days).

The NDE scores of the following students were evaluated: students of the semester before (S0) and after (S1-S3) TPC introduction who passed the NDE during a standard period of study. The semester in which the TPCs were introduced (S1) was excluded from analysis because of initial difficulties in building up a routine workflow. Students returned the TPC after their eighth semester, and the NDE took place following the tenth semester, resulting in a gap of two semesters without TPC usage.

### Clinical setting

**Fig. 2:**
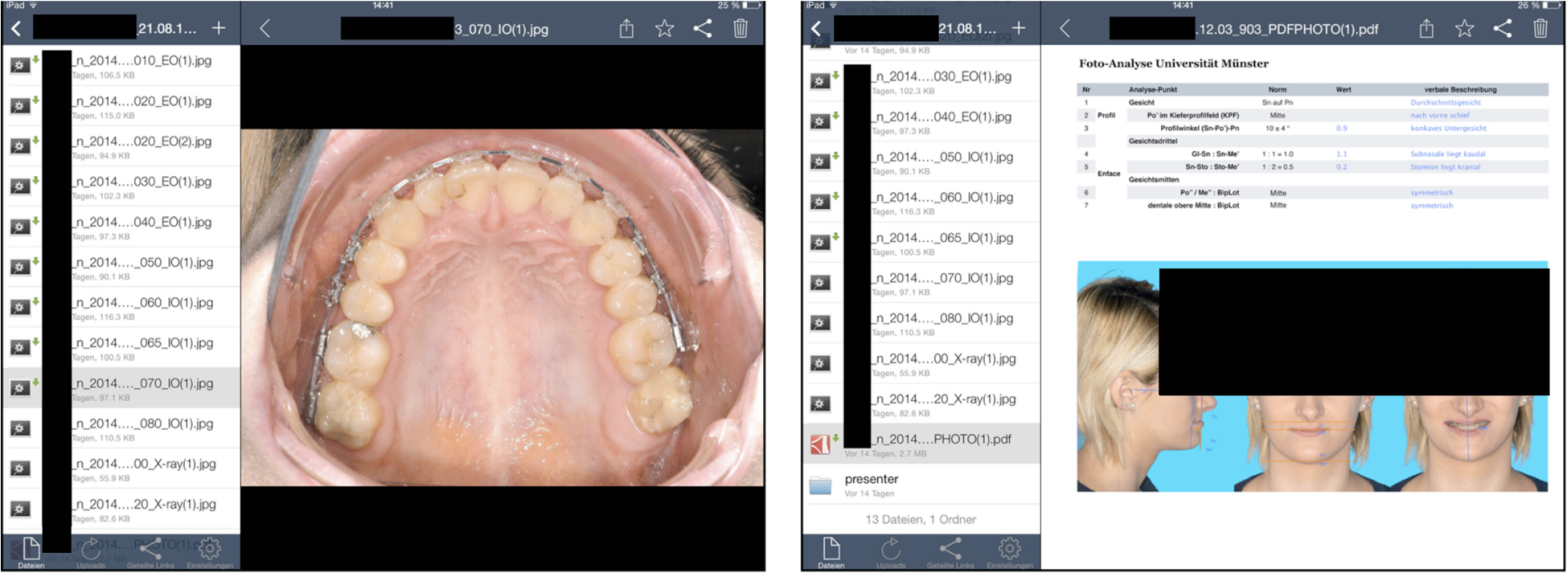
During orthodontic courses, students’ TPCs were synchronized with selected patient data via ownCloud (ownCloud GmbH, Nürnberg, Germany). Left: Consecutive intraoral photographs for reviewing treatment history. Right: Photo-analysis as PDF. All information was presented in chronological order

Students in the Department of Orthodontics are involved in routine patient treatment, in which the orthodontist-to-student ratio is 1:6 [20]. Due to the length of orthodontic treatment and the long time intervals between appointments (4 to 8 weeks), students may see the same patient only once or twice during their orthodontic education. To learn from these routine cases, it is mandatory to review the treatment history prior to seeing the patient. To achieve this, the students were given access via TPC to the extraoral and intraoral photographs, radiographs, treatment plan, and cephalometric analysis of these patients (Fig. 2).

Patient data were exported from the Orthodontic Information System via ownCloud (ownCloud GmbH, Nürnberg, Germany). Patients gave written permission to use their orthodontically related health data for teaching, which involves data storing and syncing with cloud software within the closed network of the university [21]. The TPCs were used chairside for retrieving, storing, and transmitting photos, reviewing treatment phases, viewing intraoral appliances, searching PubMed, downloading relevant articles, reading articles and textbooks, viewing educational videos (treatment sequences), making case presentations, and communicating via e-mail or social media.

### Hygienic considerations

When TPCs are used in health care, it is imperative to mitigate their risk of bacterial contamination. The TPC uses a touch screen liquid crystal display, composed of a layer of capacitive material under protective glass. Covering or touching the screen changes the charge at the point of contact. Latex gloves are conductors of electrical charge, allowing the use of the touchscreen. Nooks, crannies, and connecting ports of the device must be covered with a layer of conductive material. We have found that standard cling film fulfils this function. Therefore, chairside TPC use is permitted only with latex gloves and cling-covered screens.

### Nonclinical settings

Nonclinical settings refer to all places outside the clinic where students used the TPC for learning. These were not included in the analysis.

### Statistical analysis

Descriptive statistics were performed using the software SPSS (IBM SPSS Statistics 24 for Mac, IBM Corp, Somers, NY, USA). Chi-square tests were used to determine whether age, gender, or graduate level had any effect on the data and to test the association between the student’s expectations prior to deployment and adoption (post-usage). To assess differences in clinical versus home learning, the Wilcoxon test was used. Differences in educational benefits were tested by one-way ANOVA post hoc Tamhane.

## Results

### Survey (S2, S3)

A total of 108 students completed the survey. The male-to-female ratio was 36/72. The gender distribution in regard to the graduate student level (semester) showed no differences (chi-square; *P* = 0.984).

Of all the students, 10.1% characterized themselves as a computer beginner, 67.9% as intermediate, and 22% as advanced users, whereas 66.1% had never used the TPC before. Graduate level had no effect on computer experience (chi-square; *P* = 0.821).

As for learning it, 65.14% agreed or strongly agreed that it was easy to learn to operate the TPC. If they encountered problems, they sought help more frequently from fellow students than from staff (Wilcoxon; *P* = 0.000).

Expectations of benefits before TPC use were totally different from those experienced after taking the courses. Agreement increased for all items of the queried benefits (Table 1).

**Table 1:** Pre-use Expectations (What specific benefits did you expect from the TPC before you got it?) versus Post-use experience (What were the main benefits of using the TPC?)

	Pre-use		Post-use		*P*-value
	Mean ± SD	Median	Mean ± SD	Median	
Convenience	1.54 ± 0.5	2	3.82 ± 0.9	4	0.000
Portability	1.62 ± 0.5	2	3.80 ± 0.9	4	0.000
Ease of use	1.39 ± 0.5	1	3.64 ± 0.9	4	0.000
Helps with revision	1.16 ± 0.4	1	2.89 ± 1.1	3	0.000
Timesaving	1.20 ± 0.4	1	2.95 ± 1.2	3	0.000
Customisability	1.27 ± 0.4	1	3.30 ± 0.9	3	0.000
Keeps me in contact	1.17 ± 0.4	1	3.25 ± 1.1	3	0.000

Code: 1 = Strongly disagree; 2 = Disagree; 3 = Neutral; 4 = Agree; 5 = Strongly agree

### Clinical versus nonclinical learning (S2, S3)

Students rated the TPC slightly less useful for chairside use (2.59 ± 1.2; median = 2; disagree) than for usage during lectures (2.77 ± 1.2; median = 3; neutral), but there was no significant difference in general (Wilcoxon; *P* = 0.150). Main differences were observed between learning in the clinical versus nonclinical setting (*P* = 0.000). Students rated the TPCs more useful for learning at places outside the clinic (3.47 ± 1.0; median = 4; agree) than for learning in the clinic (2.86 ± 1.3; median = 3; neutral).

The use of PDFs and communication applications (e-mail, social media, or other video and text messengers) was different between clinical and nonclinical use. Students preferred to use these tools more frequently for learning in nonclinical settings (Wilcoxon, PDF: *P* = 0.008; communication: *P* = 0.000). Dental applications, spreadsheets, and word processing were rated lower than PDF use and communication tools (Table 2).

**Table 2:** Clinical Learning versus Nonclinical Learning Concerning Different Actions

	Nonclinical Learning		Clinical Learning		*P*-value
	Mean ± SD	Median	Mean ± SD	Median	
Word processing	3.02 ± 1.1	3	3.03 ± 1.0	3	0.907
PDF	3.73 ± 1.0	4	3.56 ± 1.0	4	0.008
Spreadsheets	2.88 ± 0.9	3	2.95 ± 0.9	3	0.203
Communication	3.85 ± 1.0	4	3.55 ± 1.0	4	0.000
Dental apps	3.27 ± 0.9	3	3.30 ± 1.0	3	0.723

Code: 1 = Strongly disagree; 2 = Disagree; 3 = Neutral; 4 = Agree; 5 = Strongly agree

**Fig. 3:**
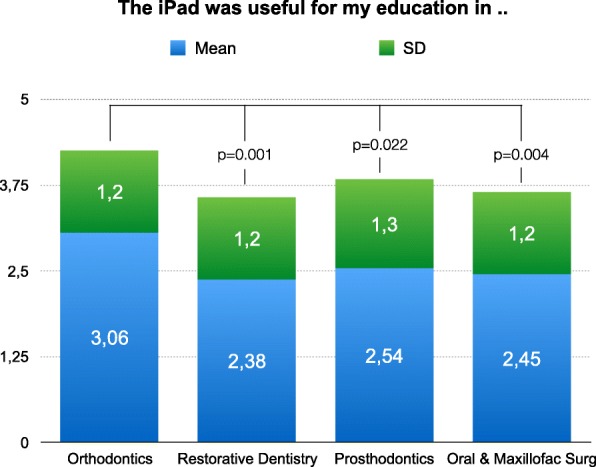
Rating of the question, "The TPC was useful for my education in .(1 = Strongly disagree; 2 = Disagree; 3 = Neutral; 4 = Agree; 5 = Strongly agree). Educational benefits differ significantly between the various dental specialties (ANOVA)

The usefulness of TPCs for general dental education was rated differently. There was no agreement between the disciplines (ANOVA; *P* = 0.000). The best rating could be found for orthodontics with a mean of 3,06. The various ratings are presented in Fig. 3.

### National Dental Examination scores (S0, S2, S3)

The NDE scores of students who attended the orthodontic course before TPC deployment (S0: *n* = 64, 32 female and 32 male, mean age 24.5 years) were compared with the scores of students who participated in the TPC one-to-one program. A total of 53 students, 27 of the first semester after TPC introduction (S2: *n* = 27, 14 female and 13 male, mean age 24.4 years) and 26 students of the second semester (S3: *n* = 26, 16 female and 10 male, mean age 24.4 years) after TPC deployment passed the NDE in a standard period of study.

The overall score in orthodontics depends on individual scores in theoretical knowledge, chairside clinical skills (specific knowledge about orthodontic devices and treatment procedures), and manual skills (construction of a removable appliance).

**Fig. 4:**
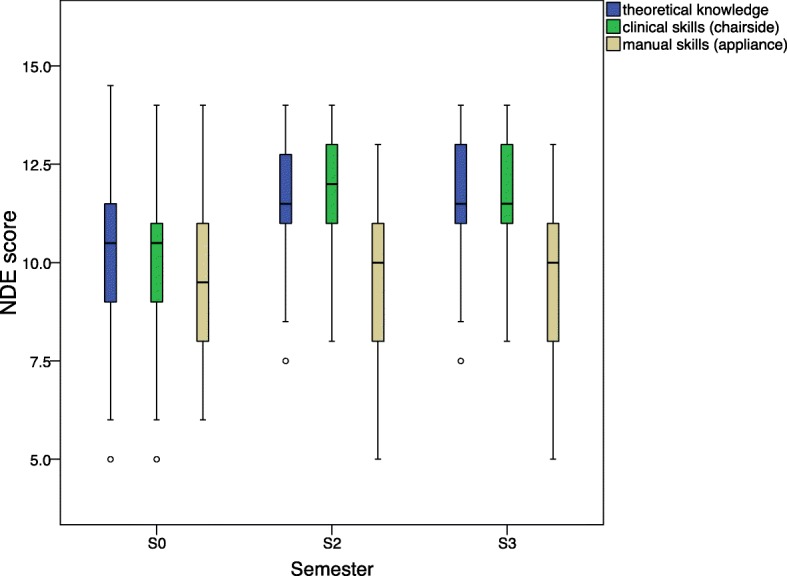
Scores according to the German grade system, varying from 0 (failed) to 13–15 points (very good), by semester (S0 before TPC introduction; S2, S3, first and second semester after TPC introduction), and by examination (theoretical knowledge, clinical and manual skills). The scores of theoretical knowledge and clinical skills improved from S0 to S2 (Wilcoxon, *P* = 0.006 resp. *P* = 0.002) and did not differ significantly from S2 to S3 (Wilcoxon, *P* = 1.000). There was no difference in manual skills between semesters (Wilcoxon, *P* = 1.000)

The scores for theoretical knowledge increased from S0 to S2 (Wilcoxon, *P* = 0.006). There was no significant difference in scores between S2 and S3 (Wilcoxon, *P* = 1.000). The same applies to the scores of the clinical skills (Fig. 4). The NDE scores after TPC introduction were significantly higher than before TPC introduction (Wilcoxon, *P* = 0.002). Nor was there any significant difference between S2 and S3 (Wilcoxon, *P* = 1.000). The scores for manual skills remained on the same level independent of TPC use (Wilcoxon, *P* = 1.000).

## Discussion

This is the first study to analyse TPC deployment in the orthodontic curriculum. It is also the first study that measured the effect of TPC learning on National Dental Examination scores.

Because of routine clinical workflows and specific dental treatment regimens, dental students in the clinical phase of education are confronted with mainly inflexible course structures. There is limited space for learners to choose the best way to acquire knowledge according to their individual strengths. Despite the various educational settings, such as laboratory, lecture hall, patient care in different departments, library, and dental simulators, the TPC provided several benefits for the students.

Before TPC introduction, students’ expectations of TPC benefits were very low. This may be associated with the participants’ computer experience. Seventy-eight per cent of all students characterized themselves as beginner or intermediate users, and 66.1% had never used the TPC. This kind of caution changed over time with using the device. There were significant improvements in all queried benefits by comparing pre-use expectations with post-use experience (Table 2). These results are in contrast to those of Luo et al., whose initially high expectations of TPC deployment in internal medicine were followed by a reduction to a more realistic level of TPC usage [12]. These authors explained their results with the Gartner hype cycle [22]. With the introduction of the TPC in 2010, Luo et al. conducted their survey on the “peak of inflated expectations [12].” Now, 7 years later, the “plateau of productivity” may be reached.

During patient dental care where the focus is on the oral cavity rather than on the display for viewing medical content, the TPC was rated less useful chairside compared with usage in the lecture hall, but not to a significant extent. Main differences were found between learning in the clinical versus nonclinical setting (*P* = 0.000). Students rated the TPC more useful for learning at home (3.47 ± 1.0; median = 4; agree) than in the clinic (2.86 ± 1.3; median = 3; neutral). In particular, viewing PDFs and communication were significantly more often used at home than in the clinic (Table 2). This is in agreement with Reynolds et al., who assessed the usability of personal digital assistants (PDAs) [23]. The authors also found better results for PDF viewing when using the device at home. In contrast, word processing on PDAs was rated better when used in the clinic. Archibald et al. found similar results for TPC use in a family medicine residency program [13]. Participants of their survey rated the TPC as very useful for typing text and for accessing teaching resources. They found that the TPC is convenient, and it helps achieve teaching goals [13]. Another study by Chase et al. also confirmed that tablets have a positive impact on learning experiences of medical student but students used the mobile learning devices more frequently outside of clinical settings [2]. A systematic review by Dunleavy et al. concluded that the included studies showed that mobile learning is equivalent or superior to traditional learning methods [3].

Students’ opinions of the educational benefits are inconsistent among the various dental specialties (Fig. 3). It could be speculated that the benefits change with the availability of digital learning content provided by the different departments.

Although self-reported measurements of improved academic performance are available, objective measurements of mobile learning’s impact on final exams or board examinations in medicine are rare [24, 25]. Baumgart et al. used the Medical Knowledge Self-Assessment Program (MKSAP), a training tool for the American Board of Internal Medicine exams, to compare scores between groups of first-year medical residents with and without TPCs. Although true board exam scores are not assessed, the authors found that only TPC use and self-rated excellent internal medicine knowledge at baseline had a significant effect on the MKSAP score [26].

Our assessment of the true final exam scores also demonstrates that TPC deployment had a significant effect on the NDE scores in orthodontics. Unsurprisingly, the scores for manual skills remained unchanged. It appears plausible that TPCs without haptic feedback are not effective in training manual dexterity. The effect of multimedia material on practical skills also remains undetermined. Different medical specialists found different effects. Physiotherapy students gained improved palpation and ultrasound skills [27], whereas dental students gained only a partial benefit for cavity removal [28]. Nor did visual methodologies during orthodontic courses have any effect on the quality of orthodontic appliances fabricated by dental students [29].

However, the fact that motor skills remained independent in NDE scores over the observational period confirmed the causal effect of TPC deployment on theoretical knowledge. Undoubtedly, not the specific device itself but the possibility to learn and communicate at any place any time with full and fast access to learning material and actual medical information increases students’ motivation and overall education quality. With this concept, the student is able to customize the course content depending on his or her learning curve.

The present study has limitations. Educational background such as previous education as dental nurse or dental technician was not investigated. The number of course participants and the time and duration of orthodontic modules were predetermined by the dental school and orthodontic curriculum. Allocating students from higher semesters to increase the sample size would bias the study because knowledge and skills vary between semesters [30]. Moreover, the number of students per semester also varies depending on their success in passing the different courses. Not all participating students passed the clinical courses up to the NDE in the standard period of study.

## Conclusions

This is the first study that analysed a one-to-one TPC program in the orthodontic curriculum and measured the effect of TPC learning on NDE scores. Students’ expectations concerning the TPC benefit in the orthodontic curriculum improved significantly by using these devices. We showed that the NDE scores of theoretical knowledge increased significantly after TPC deployment whereas the scores of motor skills remained unchanged. The results suggest that using a TPC has a positive learning effect on theoretical knowledge in orthodontics.
