# Correction to: The tablet computer’s impact on learning and National Dental Examination scores in orthodontics - a mixed-method research

**DOI:** 10.1186/s13005-019-0199-3

**Published:** 2019-06-18

**Authors:** Thomas Stamm, Irina Triller, Ariane Hohoff, Moritz Blanck-Lubarsch

**Affiliations:** Department of Orthodontics, University of Münster, Albert-Schweitzer-Campus 1, 48149 Münster, Germany

**Correction to: Head Face Med**

**https://doi.org/10.1186/s13005-019-0195-7**

Following publication of the original article [1], the editor-in-chief reported that the information regarding ethics approval was accidentally entered by the author under trial registration. A trial registration was not required for this study.
